# Natural sensitizer extracted from *Mussaenda erythrophylla* for dye-sensitized solar cell

**DOI:** 10.1038/s41598-023-40437-6

**Published:** 2023-08-24

**Authors:** Tharmakularasa Rajaramanan, Fatemeh Heidari Gourji, Yogenthiran Elilan, Shivatharsiny Yohi, Meena Senthilnanthanan, Punniamoorthy Ravirajan, Dhayalan Velauthapillai

**Affiliations:** 1 Faculty of Engineering, Western Norway University of Applied Sciences, 5020 Bergen, Norway; 2 Clean Energy Research Laboratory (CERL), Department of Physics, University of Jaffna, Jaffna, 40000 Sri Lanka; 3 Department of Chemistry, University of Jaffna, Jaffna, 40000 Sri Lanka

**Keywords:** Energy science and technology, Materials science, Optics and photonics

## Abstract

In this study, a natural dye from the flowers of *Mussaenda erythrophylla* extracted separately in ethanol and de-ionized water was employed as a photosensitizer in DSSCs. The quantitative phytochemical analyses were performed on both extracts. The existence of flavonoids (anthocyanin) and chlorophyll a pigments in the ethanol extract of the dye was confirmed by the UV–Visible spectroscopy. The stability study performed on the said ethanol extract confirmed that the dye extracted in ethanol was stable in the dark and did not degrade for nearly 50 days. The presence of the dye molecules and uniform adsorption of them on the P25-TiO_2_ surface were confirmed by fourier transform infrared spectroscopy and atomic force microscopy, respectively. Moreover, the influence of dye concentration and pH on the optical properties of the dye was also studied. The natural dye extracted in ethanol was employed in DSSCs, fabricated by utilizing the said dye sensitized P25-TiO_2_ photoanodes, ${I}^{-}$/${I}_{3}^{-}$ electrolyte, and Pt counter electrode. Photovoltaic performances of the fabricated devices were determined under simulated irradiation with the intensity of 100 mWcm^–2^ using AM 1.5 filter. The device fabricated with the P25-TiO_2_ photoanode sensitized by the dye extracted in ethanol at pH = 5 exhibited the best power conversion efficiency (PCE) of 0.41% with the J_SC_ of 0.98 mAcm^–2^ which could be attributed to the optimum light absorption in the visible region of solar spectrum by the chlorophyll a and anthocyanin molecules in the extracted natural dye.

## Introduction

Dye-sensitized solar cells (DSSC) have gained worldwide attention for many years due to low production cost and environmentally friendly operation. The operation principle of DSSC is similar to the photosynthesis, a natural process. Here, the device is capable of generating energy by converting the absorbed Sunlight into electrical energy. Generally, a DSSC is composed of a mesoporous metal oxide semiconductor, a dye sensitizer, an electrolyte containing iodide and triiodide ions and a counter electrode^1^. In DSSC, sensitizers play a key role in harvesting the sunlight and then transforming it into electrical energy. Numerous metal complexes and organic dyes have been synthesized and utilized as sensitizers. Yet, ruthenium based synthetic organic dyes are found to be effective sensitizers. By far, the highest efficiency of over 11% has been reported for DSSCs sensitized by Ru based N719 dye^2^. However, the preparation routes for metal complexes are often based on multi-step procedures involving tedious and expensive chromatographic purification procedures^3^. It is anticipated that replacing synthetic organic dyes with natural pigments, such as chlorophyll and anthocyanin, could resolve the above limitations as they can be easily extracted from the fruits, leaves, roots and flowers of plants.

Generally, many of the plant parts contain chlorophyll and anthocyanin pigments. Chlorophyll is the most abundant pigment in green plants and each chlorophyll molecule possesses a Mg^2+^ ion surrounded by four pyrrole rings, one of which is bonded to a phytol tail^4^. Chlorophyll molecules are described as photoreceptors due to their light absorbing property. There are two types of chlorophyll, namely chlorophyll a and chlorophyll b, which differ in their structures at the C3 position of one of the pyrrole rings. The C3 position of the said pyrrole ring in chlorophyll b contains a formyl (–CHO) side chain whereas a methyl (–CH_3_) group is present at the same position in chlorophyll a^5^. Due to the presence of different substituents, the chlorophyll a and chlorophyll b molecules exhibit varied light absorption properties. Hence, chlorophyll absorbs light in a wide wavelength range corresponding to blue, red and violet regions of the visible spectrum^6^. Anthocyanin is another pigment responsible for the variety of colours in petals of flowers and fruits. Employing anthocyanin as dye for DSSC leads to absorption of light in the blue-green region of the solar spectrum^7^ and the carbonyl and hydroxyl groups present in anthocyanin molecules demonstrate efficient anchoring to the TiO_2_ surface (photoelectrode), thus enabling an effective electron injection mechanism in DSSC^8^.

Commonly, natural dyes contain either chlorophyll or anthocyanin and rarely contain both. Studies focusing on enhancing PV performance of the fabricated devices with dye mixtures comprising of both pigments have been reported. Wongcharee et al.^9^ fabricated three different types of DSSCs with natural dyes extracted from rosella and blue pea and a mixture of the said dye extracts. The light absorption spectrum of the dye mixture showed peaks corresponding to the individual natural pigments present in rosella and blue pea. However, the dye mixture adsorbed on TiO_2_ did not exhibit synergistic light absorption and photosensitization effect compared to the individual constituent dyes^9^. In a different study, Sengupta et al*.*^10^ has reported that a mixture of chlorophyll and betalain dyes, extracted from fresh spinach leaves and beetroots respectively, achieves photovoltaic performance of the device upto 0.29% due to light absorption in a wider range of solar spectrum^10^. In a separate study, Park et al*.*^11^ has demonstrated mixing the dyes, extracted from the flowers of *Gardenia Jasminoide Elli* with two different colours (yellow and blue), results in widening the light absorption wavelength compared to the individual dyes, thus improving the PV performance of the fabricated device^11^.

For the present study, we have chosen the flower of *Mussaenda erythrophylla*, which contains both chlorophyll and anthocyanin, as a natural sensitizer for DSSCs. *Mussaenda erythrophylla* is a rambling shrub, grows best in warmly temperate or subtropical regions and is semi deciduous in cooler parts^12^. The flower of the plant possesses much smaller flowers in the centre and is available in a wide variety of colours, including red, rose, white and pale pink. The flowers bloom for most of spring through summer. Since this plant is widely found in Sri Lanka, the suitability of the dye extracted from the flowers of the plant in DSSC application was investigated. The study involved extraction of the dye from the flowers of *Mussaenda erythrophylla*, its phytochemical analysis, optical, structural and electrochemical characterizations and evaluation of PV performance of the corresponding devices.

## Experimental

### Materials

Absolute ethanol (> 99%), Triton X-100 (laboratory grade), di-tetrabutylammonium *cis*-bis (isothiocyanato) bis (2,2’-bipyridyl-4,4’-dicarboxylato) ruthenium (II) dye (N-719, 95%), acetonitrile (gradient grade), *tert*-butyl alcohol (≥ 99.7%) and titanium dioxide nanopowder (21 nm primary particle size, ≥ 99.5% trace metals basis) were purchased from Sigma–Aldrich, Oslo, Norway. Acetylacetone (≥ 99.5%) was purchased from Fluka Analytical, Munich, Germany. All the materials were used without further purification unless otherwise stated.

### Characterization

The optical absorbance spectra were recorded using Shimadzu 1800 scanning double beam UV–Visible spectrophotometer. The structural properties of the dye coated films were studied by fourier transform infrared spectroscopy (Thermo Scientific™ Nicolet™ iS5 FTIR spectrometer) and atomic force microscopy (AFM-Park XE7, in 1 × 1 µm scan area). pH of the prepared dye solutions was measured using Eutech pH 700 m. The photovoltaic performance of the fabricated devices with effective area of 0.25 cm^2^ was studied using Keithley-2400 source measurement unit under simulated irradiation by 150 W Xe lamp with an intensity of 100 mWcm^−2^ and AM 1.5 filter (Peccell-PEC-L12, Kanagawa, Japan). All electrochemical studies were carried out using Bio Logic SP-150 potentiostat.

### Methodology

#### Natural dye extraction

**Figure 1:**
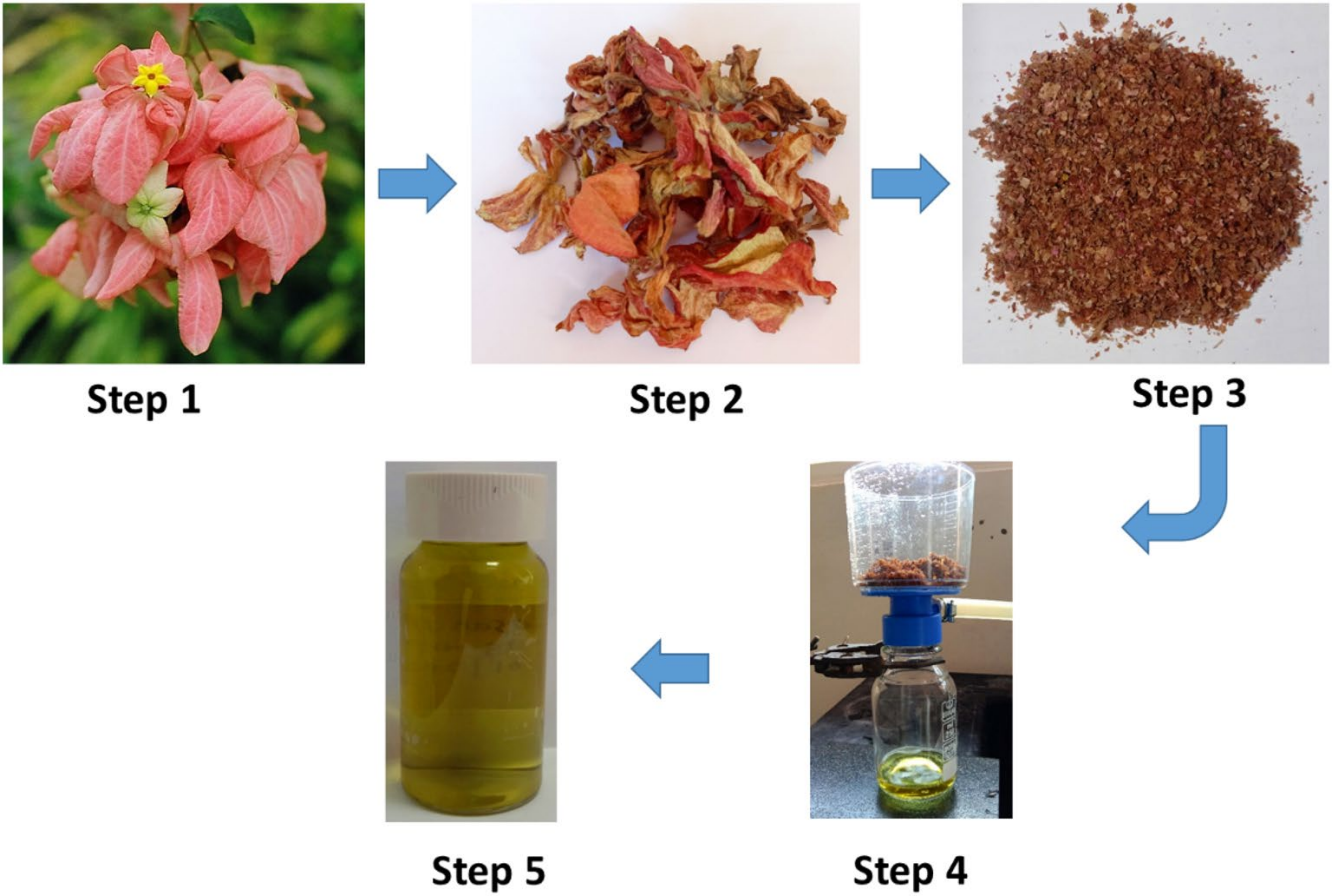
Schematic representation of the procedures followed during extraction of the natural dye.

The natural dye was extracted from the flower of *Mussaenda erythrophylla*. Initially, the fresh flowers of *Mussaenda erythrophylla* were collected from the Thirunelvely premises of University of Jaffna, Jaffna, Sri Lanka and washed with tap water followed by de-ionized water (DI-water) to remove the dust particles (Step 1). Then, the cleaned flower petals were allowed to dry at a muffle furnace for 2 days at 60 °C (Step 2). The dried petals were crushed into powder using an electrical blender (Step 3). 1 g of the dried petal powder was soaked in 10 mL of two different solvents (ethanol and DI-water) separately for 24 h. Then, the solutions were filtered separately using Whatman No.1 filter paper (Step 4). The individual filtrates (natural dye samples) were stored in airtight containers at room temperature and used as sensitizers without further purification (Step 5) (Fig. 1). All procedures involving plant materials were performed in compliance with the relevant ethical standards and institutional and/or national guidelines/regulations/legislation.

#### Phytochemical analysis

The natural dye samples, extracted separately in ethanol and DI-water, were heated at 60 °C to remove the unwanted volatile substances and the resultant residues were stored appropriately. The said residues of 162 mg from ethanol extract and 160 mg from DI-water extract were again dissolved separately in 20 mL of the respective solvents and the phytochemical analysis was carried out by adopting the standard procedures (Table S1) stipulated elsewhere^13,14^. The results of phytochemical analysis are given in Table 1 and Fig. S1.

**Table 1:** Phytochemical analysis of *Mussaenda erythrophylla* flower extracted in ethanol and DI-water.

Phytochemical component	Ethanol extract	DI-water extract
Terpenoids	** + **	** + + **
Flavonoids	** + + **	**–**
Glycosides	** + **	** + + **
Alkaloids	** + **	** + + **
Phenols	** + + **	** + **
Quinones	** + + **	** + + **
Coumarins	** + + **	** + **

#### Device fabrication

The Fluorine doped Tin Oxide (FTO) coated glass substrates with surface resistivity of 7.5 Ωcm^–2^ were cleaned in ultrasonic bath for 10 min by treating sequentially with soap water, DI-water and ethanol. 100 mg of P25-TiO_2_ was ground with DI-water, acetylacetone and Triton X-100 using an agate motor with pestle to make semisolid TiO_2_ paste. Subsequently, the prepared TiO_2_ paste was coated on the previously cleaned FTO glass by doctor blading method and calcinated at 500 °C for 30 min to obtain TiO_2_ coated thin films. Then, the said films were soaked separately in the natural dye samples extracted in ethanol and DI-water for 12 h. Afterwards, the dye coated thin films were rinsed with the respective solvents and dried. The platinum (Pt) coated glass substrate was assembled with the individual dye-coated photoanode as the counter electrode. Finally, a small amount of ${I}^{-}$/${I}_{3}^{-}$ electrolyte was injected in between the dye coated photoanode and Pt counter electrode to complete the DSSC fabrication.

## Results and discussion

### Phytochemical analysis

The dried flower petal powder of *Mussaenda erythrophylla* was soaked in ethanol and DI-water separately and the corresponding natural dye samples were extracted and subjected to quantitative phytochemical analysis which revealed the presence of phytochemicals as displayed in Table 1.

As per Table 1, both (ethanol and DI-water) extracts of *Mussaenda erythrophylla* flower contain terpenoids, glycosides, alkaloids, phenols, quinones, and coumarins in different quantities. Also, the above phytochemical analysis reveals that flavonoids are absent in the DI-water extract although presence of flavonoids in the flowers of *Mussaenda erythrophylla* has been reported in the literature^15^. Anthocyanin is one of the pigments responsible for the colour of flower petals and belongs to the family of flavonoids^16^. It consists of phenolic-OH group (hydroxyl group bonded directly to an aromatic hydrocarbon ring) and frequently occurs in plants as glycosides (bound to sugar groups)^17^. Since the phytochemical analysis of the DI-water extract of *Mussaenda erythrophylla* flower demonstrates the presence of phenols and glycosides, it could be assumed that modified anthocyanin pigments may be found in the said extract.

### Optical characterization

#### UV–Visible spectroscopic analysis

**Figure 2:**
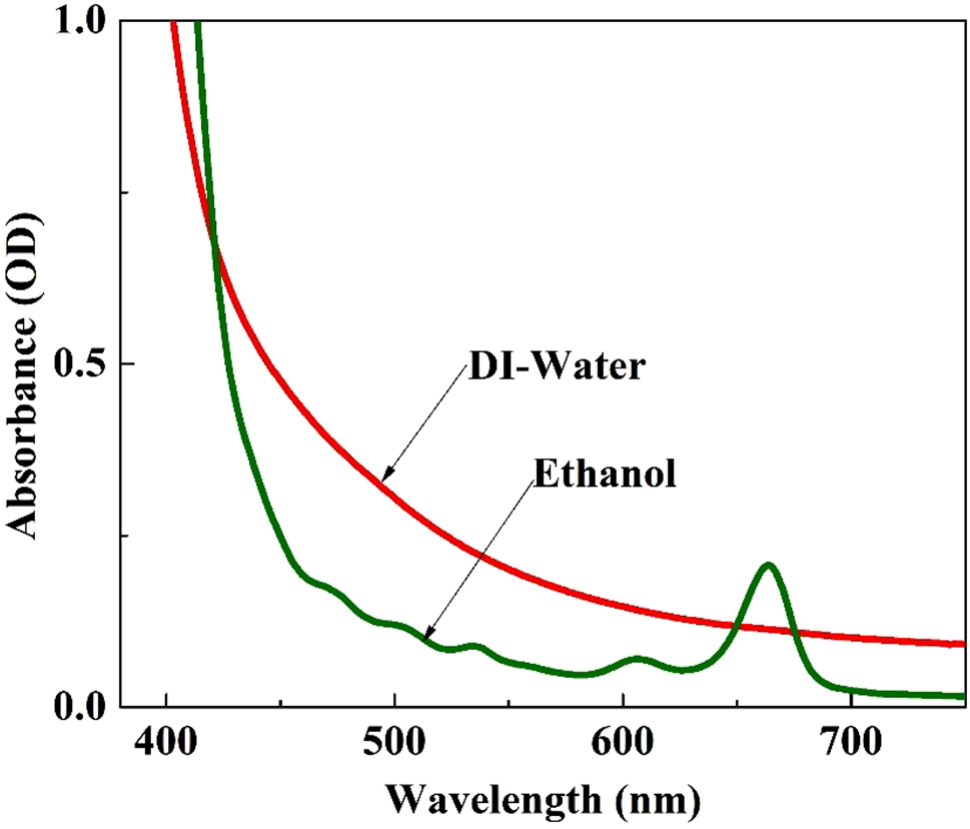
UV–Visible spectra of the dye extracted from *Mussaenda erythrophylla* flower using ethanol and DI- water.

The optical properties of both dye extracts and the corresponding dye coated TiO_2_ films were analyzed by UV–Visible spectroscopy. Prominent optical properties were observed for the dye extracted in ethanol compared to DI-water. The peak observed at 533 nm for the ethanol extract as shown in Fig. 2 corresponds to the light absorption by anthocyanin molecules^18^ which is in good agreement with the phytochemical study. In addition, an intense absorption peak at 665 nm corresponding to the n to π* transition in chlorophyll a molecule was observed for the ethanol extract^19,20^. The observed absorption peak of chlorophyll a is dominant over the absorption peak of anthocyanin. Overall, the UV–Visible spectrum of the ethanol extract confirms that chlorophyll a and anthocyanin pigments are successfully extracted in ethanol from the *Mussaenda erythrophylla* flower petals.

But, the DI-water extract did not show any peak in the regions corresponding to anthocyanin and chlorophyll a absorptions. Hence, it is expected that the natural dye extracted from *Mussaenda erythrophylla* flower in ethanol would exhibit better PV performance than the dye extracted in DI-water.

#### Dye stability and sensitization

**Figure 3:**
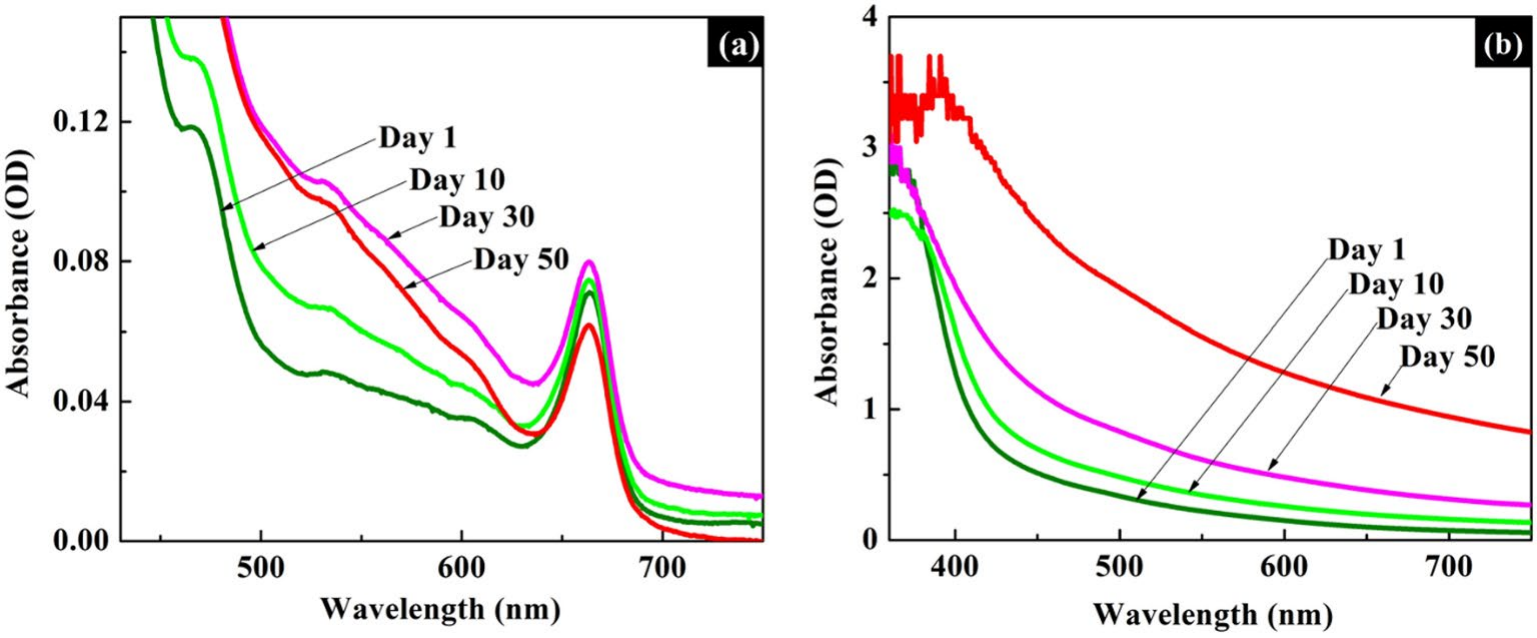
Time dependent UV–Visible spectra of natural dye extracted from *Mussaenda erythrophylla* flower in (**a**) ethanol (**b**) DI-water.

A dye with long term stability is preferred in DSSC applications. Usually, the stability of natural dyes decreases with time as they undergo biodegradation and photo-oxidation. Hence, stabilities of the natural dye samples extracted from *Mussaenda erythrophylla* flower in ethanol and DI-water were determined in the present study by keeping the respective dye solutions in the dark at room temperature and measuring their light absorbance intensities, using UV–Visible spectrophotometer, periodically for 50 days (Fig. 3).

The time dependent UV–Visible spectrum of the dye extracted in ethanol did not show any significant change with time, except a reduction in intensity of the peak at 665 nm corresponding to chlorophyll a. In contrast, the said spectrum of the dye extracted in DI-water exhibited increased light scattering with time which may be due to fungal growth. Hence, the stability study suggests that the dye extracted in ethanol is more stable than the dye extracted in DI-water.

When employing a dye sensitizer in photovoltaics, optimizing its sensitization duration is essential. In order to accomplish this, the TiO_2_ coated films were dipped in ethanol and DI-water extracts of *Mussaenda erythrophylla* flower separately and their light absorption properties were studied at regular time intervals using UV–Visible spectrophotometer.

**Figure 4:**
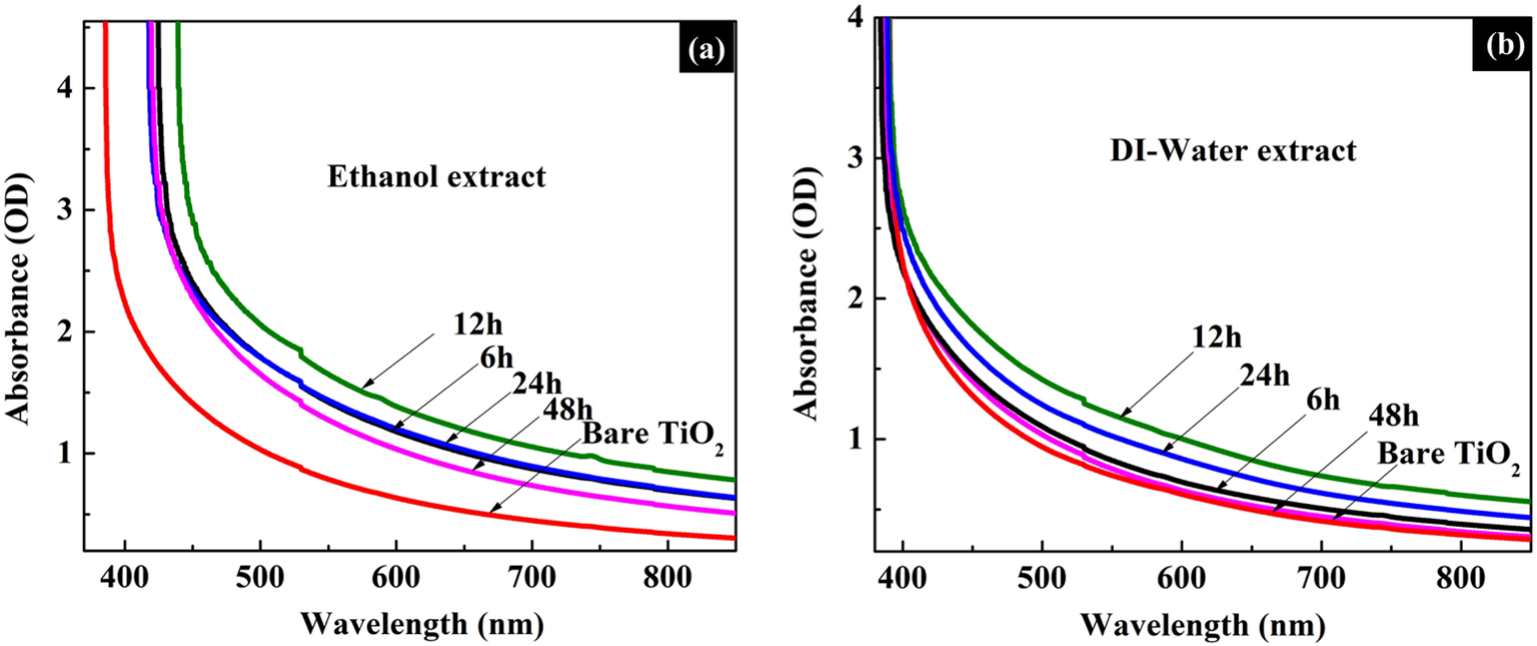
Time dependent UV–Visible spectra of TiO_2_ films dipped in the dye extracted from *Mussaenda erythrophylla* flower in (**a**) ethanol (**b**) DI-water.

As illustrated in Fig. 4, a monolayer of dye molecules formed on the TiO_2_ surface upon 12 h of sensitization in both extracts. Interestingly, TiO_2_ films coated with the dye samples extracted in ethanol and DI-water showed similar peaks in the UV–Visible spectra. Further, greater light absorption capabilities were exhibited by both dye coated TiO_2_ films compared to the bare TiO_2_ film. In addition, UV–Visible spectroscopic measurements of the dye solutions, taken before and after dipping the TiO_2_ films in the said dye solutions, demonstrated a reduction in their light absorption capabilities, thus confirming that dye molecules had been adsorbed on the TiO_2_ surface while dipping (Fig. S2).

### Structural characterization

#### FTIR spectroscopic analysis

**Figure 5:**
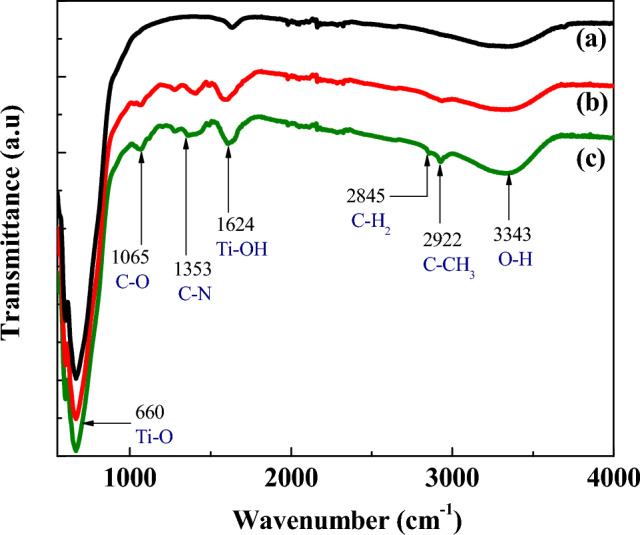
FTIR spectra of (a) bare TiO_2_ (b) TiO_2_ sensitized with dye extracted in DI-water and (c) TiO_2_ sensitized with dye extracted in ethanol.

It is noteworthy to mention here that strong anchoring between the functionalized groups of the dye sensitizers and the surface of the TiO_2_ nanoparticles is vital for injection of electrons from the excited state (ES) energy level of the dye molecule into the conduction band (CB) of TiO_2_ molecule, thus producing highly efficient DSSCs. The FTIR spectroscopic analysis is widely used to investigate such anchoring in dye coated TiO_2_ films. In the present study, bare and dye coated TiO_2_ nanoparticles were detached from the corresponding FTO glasses and subjected to FTIR measurements within the 400 to 4000 cm^–1^ wavenumber range and the results are illustrated in Fig. 5.

The FTIR spectra of dye coated TiO_2_ films indicate the presence of the same sensitizers in both dye extracts with a few shifts in wavenumber and trivial deviations in the transmittance level (Fig. 5).

As displayed in Table 2, the IR spectrum of the dye extracted in ethanol showed peaks at 3343, 2922, 2845, 1353 and 1065 cm^−1^ corresponding to stretching vibrations of O–H, C–H (asymmetric), C–H (symmetric), C–N and C–O groups, respectively while the C–N and C–H (symmetric) stretching vibrations were not observed in the IR spectrum of the dye extracted in DI-water. It should be noted that the porphyrin ring in chlorophyll a molecule is responsible for the appearance of peak corresponding to C–N stretching vibration in the IR spectrum; hence it is confirmed that the dye extracted in ethanol contains chlorophyll a molecules which is in consistent with the findings of UV–Visible spectroscopic analysis (Fig. 2).

**Table 2:** IR absorptions of functional groups in *Mussaenda erythrophylla* flower dye adsorbed on TiO_2_ films.

Functional group	Typical absorption range (cm^−1^)	Type of vibration	Absorption peak of TiO_2_ sensitized with dye extracted in ethanol (cm^−1^)	Absorption peak of TiO_2_ sensitized with dye extracted in DI-water (cm^−1^)	Ref
Ti–O–Ti	400–800	Stretching	660	660	^21^
C–O (Ether)	1000–1300	Stretching	1065	1065	^22^
C–N (Amine)	1080–1360	Stretching	1353	–	^23^
C–H (Alkane)	2820–2850	Stretching (symmetric)	2845	–	^24^
C–H (Alkane)	2850–3000	Stretching (asymmetric)	2922	2936	
O–H (Phenol)	3200–3600	Stretching	3343	3343	

**Figure 6:**
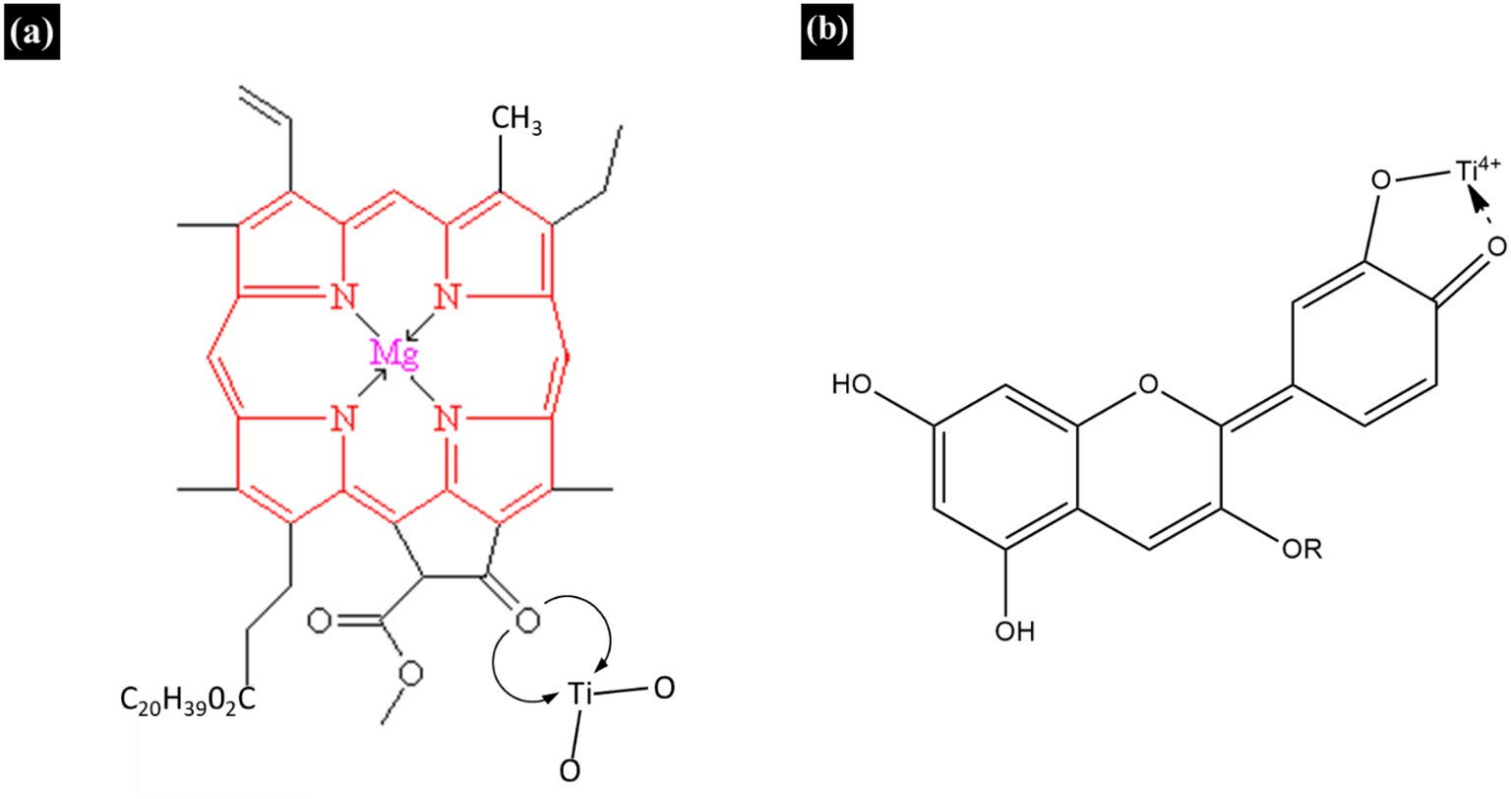
Bonding of TiO_2_ molecule with (**a**) chlorophyll a molecule and (**b**) anthocyanin molecule.

As illustrated in Fig. 6, chlorophyll a molecule attaches to the TiO_2_ surface through the carbonyl group of porphyrin ring by C=O⋅ ⋅ ⋅ TiO_2_ coordination^25^ and anthocyanin molecule anchors to the TiO_2_ molecule with its carbonyl and hydroxyl groups^26^. However, the presence of such anchoring between the TiO_2_ surface and the sensitizers (chlorophyll a and anthocyanin) could not be confirmed from the obtained IR spectral data.

#### Atomic force microscopic (AFM) analysis

The distribution of dye molecules on the TiO_2_ surface influences the PV performance of the DSSC. In the present study, distribution of dye in the prepared dye coated TiO_2_ films was analyzed and compared with the bare TiO_2_ film by AFM.

**Figure 7:**
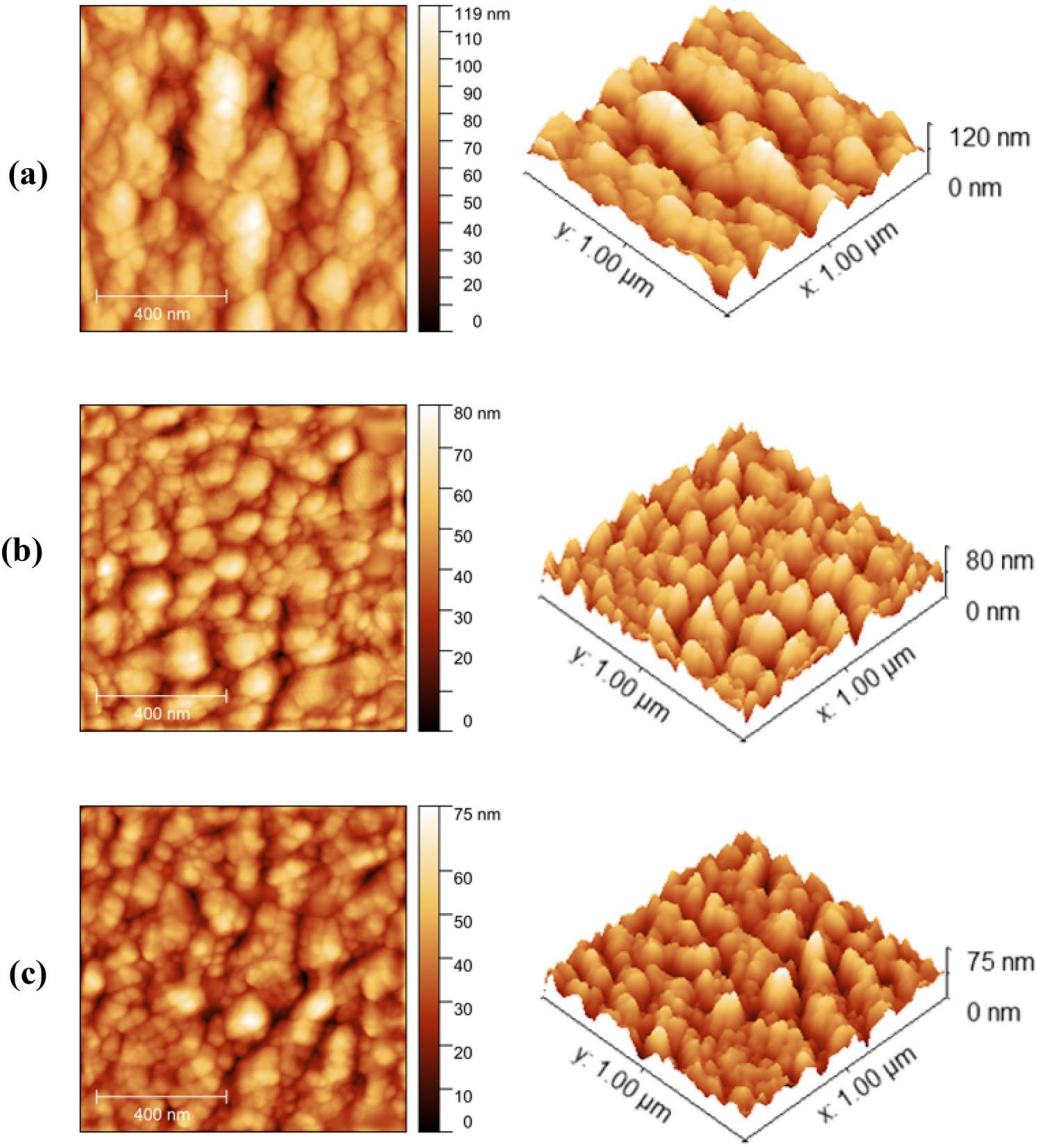
2D and 3D Topographic images of (**a**) bare TiO_2_ film (**b**) TiO_2_ film sensitized with dye extracted in DI-water and (**c**) TiO_2_ film sensitized with dye extracted in ethanol.

As shown in Fig. 7, a high degree of roughness was observed on the surface of bare TiO_2_ film. The root mean squares of roughness of bare TiO_2_, TiO_2_ sensitized with dye extracted in DI-water and TiO_2_ sensitized with dye extracted in ethanol were found to be 16.22, 10.46 and 9.46 nm, respectively. The reduction in TiO_2_ surface roughness upon sensitization with the dye could be attributed to filling of pores on the TiO_2_ surface by the said dye molecules. Also, the uniform distribution of colour in the images of dye sensitized TiO_2_ films indicates that the dye molecules are uniformly dispersed over the entire TiO_2_ surface.

### Electrochemical studies

Since the dye extracted from *Mussaenda erythrophylla* flower in ethanol demonstrated more suitable optical and structural characteristics for PV application than the dye extracted in DI-water, the cyclic voltammetry (CV) performance of the DSSC fabricated with the said dye sensitizer extracted in ethanol was analyzed. The CV was measured by employing a nickel foam dipped in the dye extracted in ethanol, Pt electrode, Ag_(s)_/AgCl_(s)_ electrode, and 2 M KOH_(aq)_ as working electrode, counter electrode, reference electrode and electrolyte respectively^27^ and CV of bare Ni foam was also measured for comparison.

**Figure 8:**
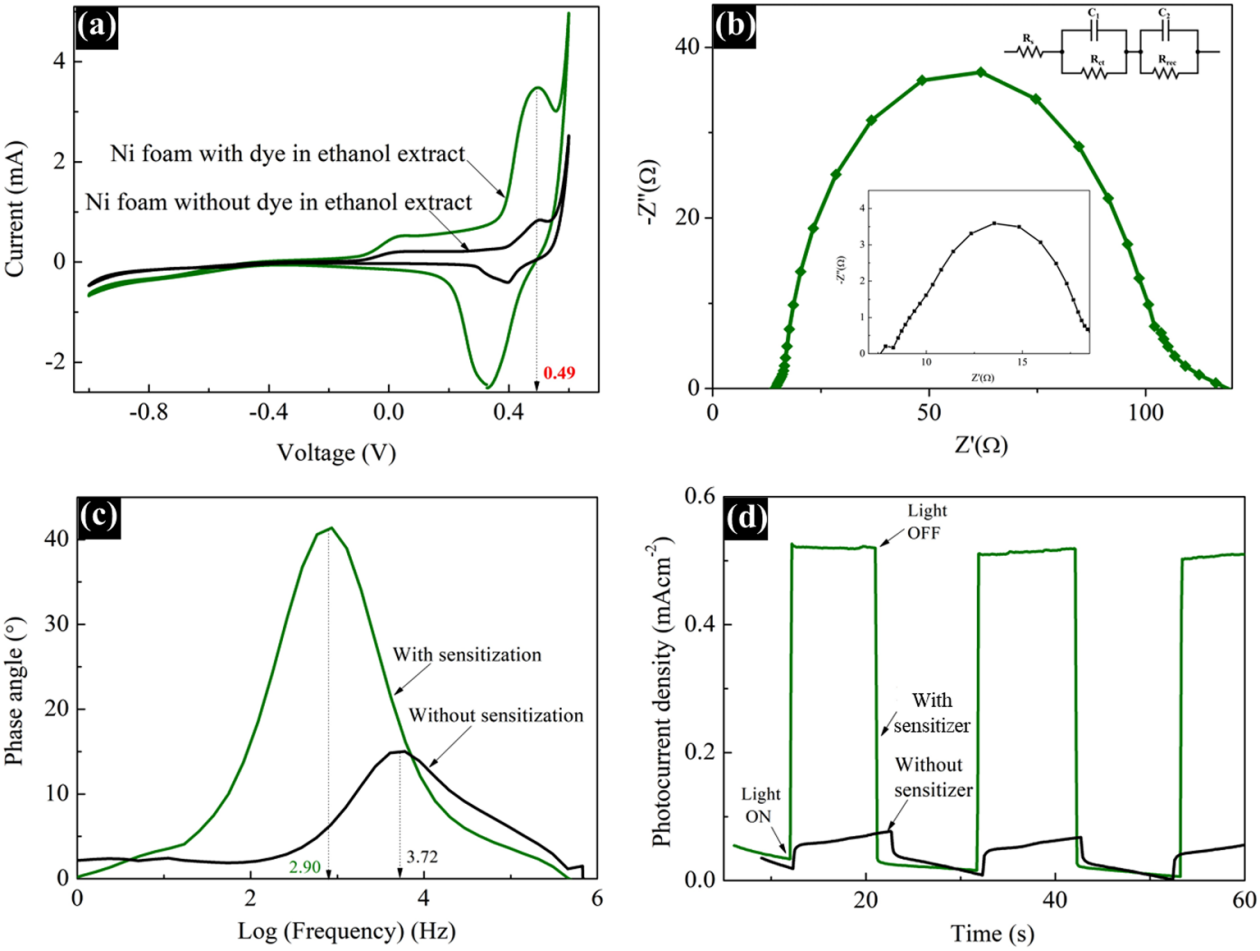
(**a**) Cyclic voltammograms of Ni foam with and without the dye in ethanol extract (**b**) Nyquist plots (**c**) Bode plots and (**d**) Transient photocurrent—Time (J_photo_–t) profile of DSSCs with and without sensitization by the dye in ethanol extract.

As shown in Fig. 8a, the oxidation $({E}_{oxd}^{onset}$) onset potential of the ethanol extract was determined from the intersection of tangent between the rising current and the baseline charging current of the respective CV curve. The calculated value for ${E}_{oxd}^{onset}$ was 0.49 eV. Further, energies of the highest occupied molecular orbital (${E}_{HOMO}$) and the lowest unoccupied molecular orbital (${E}_{LUMO}$) were calculated using the following equations as reported elsewhere, where a correction factor of 4.4 eV was incorporated for the Ag_(s)_ /AgCl_(s)_ reference electrode^28–30^ and the optical band gap (${E}_{g}^{opt}$ = 2.20 eV) was obtained from Tauc plot (Fig. S3).

$${E}_{HOMO}=-\left({E}_{oxd}^{onset}\right)+\left(-4.4\right),$$

$${{E}_{LUMO}=E}_{HOMO}+{E}_{g}^{opt}.$$

The calculated values for ${E}_{HOMO}$ and ${E}_{LUMO}$ were – 4.89 and – 2.69 eV respectively. Since the literature value for conduction band (CB) energy of TiO_2_ is nearly – 4.3 eV^31^, obviously the energy of LUMO of the dye extracted from *Mussaenda erythrophylla* flower in ethanol (– 2.69 eV) is greater than the CB energy of TiO_2_. Therefore, injection of electrons from the photoexcited dye molecule to the CB of TiO_2_ molecule is possible. Moreover, regeneration of the oxidized dye sensitizer during the operation of DSSC is possible when HOMO of the dye is lower than redox potential of the iodide/triiodide couple. In this study, energy in the HOMO (– 4.89 eV) of ethanol extract is slightly lower than the redox potential of the iodide/triiodide couple (– 4.8 eV)^32^ compare to general metal complex sensitizers. This may be the reason it can’t achieve the PCE of the device comparable to the synthetic metal complex-based dyes.

Further, the charge transport properties at the interface of the fabricated DSSC were evaluated by electrochemical impedance spectroscopy (EIS)^33–35^. The EIS measurements were recorded at the frequency range from 10^–2^ to 10^6^ Hz with 0.7 V applied bias voltage under dark condition. The impedance at each interface was determined, after fitting the EIS data in the ZView software, in terms of an appropriate equivalent circuit which is inserted in Fig. 8b. The device, sensitized by the dye in ethanol extract, exhibited series resistance at the FTO/TiO_2_ interface (R_s_), charge transport resistance at the electrolyte/counter electrode interface (R_ct_), and recombination resistance at the TiO_2_/dye/electrolyte interface (R_rec_) along with the time taken for recombination (${\mathrm{T}}_{{\varvec{e}}}$) which were quantified as 14.63 Ω, 1.60 Ω, 85.52 Ω and 54.88 ms. Moreover, the device without the dye sensitizer demonstrated R_rec_ and ${\mathrm{T}}_{{\varvec{e}}}$ values of 2.29 Ω and 42.78 ms respectively (inserted Fig. 8b and c). The very low R_rec_ value for the device without dye sensitizer indicates a high rate of charge recombination.

To understand the photovoltaic response stability of the device sensitized by the natural dye in ethanol extract with time, the transient photocurrent—time (${\mathrm{J}}_{\mathrm{photo}}-\mathrm{t})$ profile was recorded and is displayed in Fig. 8d. The photocurrent was provided at regular time intervals in response to a light on–off sequence produced by opening and closing a mechanical shutter manually that blocks the light beam (1 sun illumination). The curve representing the DSSC with the natural dye sensitizer showed the stability of photocurrent with no significant loss or decay during the illumination period of 60 s compared to the device without sensitizer. This shows that the dye regeneration process is very fast and analogous to injection of charge carriers to the CB of TiO_2_ by the excited dye molecules. All the electrochemical studies suggest that this natural dye is a potential candidate to be employed as a sensitizer in DSSC application.

### PV performance

#### Influence of solvents employed for dye extraction

**Figure 9:**
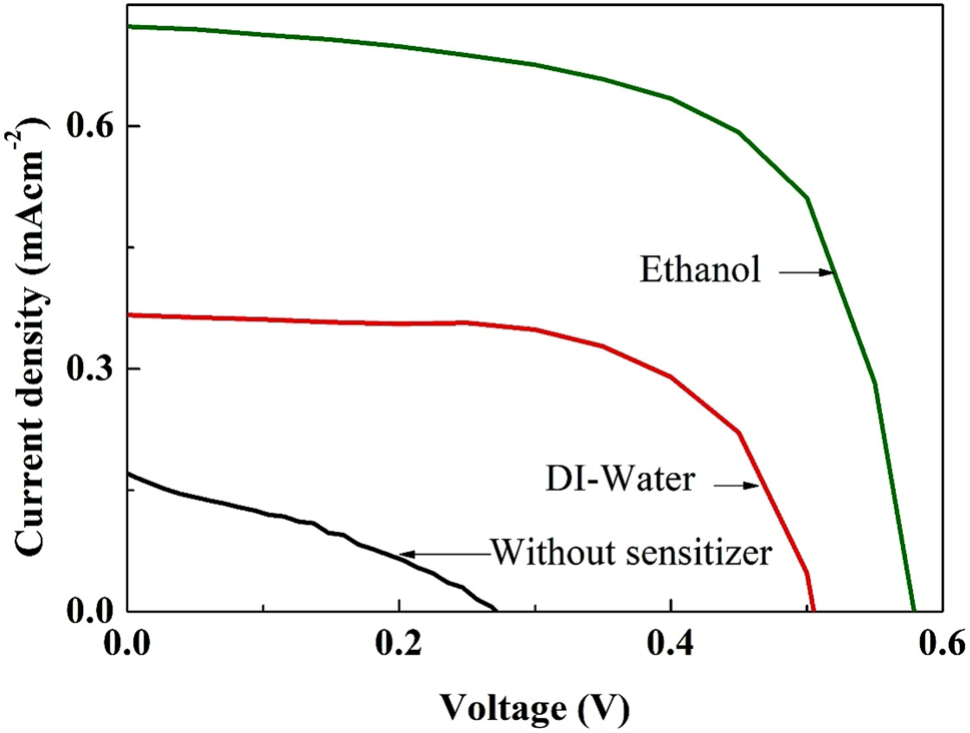
Photovoltaic performances of DSSCs with photoanodes sensitized by the natural dye extracted in ethanol and DI-water under illumination intensity of 100 mWcm^*–*2^ with AM 1.5 filter.

The PV performances of the optimized photoelectrodes were analyzed under the illumination intensity of 100 mWcm^−2^ with Air Mass (AM) 1.5 filter and effective device area of 0.25 cm^2^. Figure 9 shows J-V curves of the P25-TiO_2_ based DSSCs sensitized with the natural dye extracted from *Mussaenda erythrophylla* flower in ethanol and DI-water and the control device. The power conversion efficiency (η) expression can be written as follows:

$$\eta =\frac{{J}_{sc} \times {V}_{oc} \times FF}{{P}_{in}} \times 100 \%.$$

The short circuit current density, ${J}_{SC}$ of the device, is defined as the current density passes through the device when the applied voltage is zero. Open circuit voltage, ${V}_{OC}$, is the voltage developed in the device when the current passes through the device is zero. ${P}_{in}$ is the intensity of light irradiation and *FF* is the fill factor of the device which is defined as

$$FF = \frac{{J}_{m} \times {V}_{m} }{{J}_{sc} \times {V}_{oc}},$$

where ${J}_{m}$ is the current density at peak power and ${V}_{m}$ is the voltage at peak power. The values of all photovoltaic parameters obtained from the J-V curves are summarized in Table 3.

**Table 3:** Photovoltaic parameters of DSSCs with photoanodes sensitized by the natural dye extracted in ethanol and DI-water under illumination intensity of 100 mWcm^–2^ with AM 1.5 filter.

Sensitizer	J_SC_ (mAcm^–2^)	V_OC_ (V)	FF	η (%)
Dye in DI-water extract	0.37	0.50	0.63	0.12
Dye in ethanol extract	0.72	0.58	0.64	0.27
None	0.17	0.27	0.41	0.02

As per Table 3, relatively higher J_SC_, V_OC_, FF and η values of 0.72 mAcm^–2^, 0.58 V, 0.64 and 0.27% respectively were observed for the device sensitized by the natural dye extracted in ethanol while the same device structure sensitized by N719 dye (a Ru-based dye) exhibited the PCE (η) of 5.15% (Fig. S4). It was noted that the V_OC_ and FF had shown slight enhancements while the J_SC_ had demonstrated a significant improvement in the device with photoanode sensitized by the dye in ethanol compared to the photoanode with the dye in DI-water sensitization. A two-fold increment in the J_SC_ (from 0.37 to 0.72 mAcm^–2^) and the efficiency (from 0.12 to 0.27%) was observed for the device with the photoanode sensitized by the dye in ethanol extract compared to the device sensitized by the dye in DI-water extract. Moreover, the control device (without sensitizer) exhibited poor photovoltaic performance which was 14 times lesser than the PV performance of the best device in the present study. The following factors may have contributed to the said observations: firstly, better solubility of natural pigments (chlorophyll a and anthocyanin) found in *Mussaenda erythrophylla* flower in ethanol which could have prevented aggregation of the pigment molecules and led to their uniform dispersion on the TiO_2_ surface as evidenced by the AFM studies; secondly, more light harvest in the visible region of solar spectrum by the pigment molecules extracted in ethanol as shown in Fig. 2 that resulted in increased electron transfer from the LUMO of the excited dye to the TiO_2_ conduction band. Furthermore, the observed photovoltaic performances of the said devices were found to be retained for 24 h (Fig. S5 and Table S2).

#### Influence of dye concentration and pH

The influence of dye concentration and pH of the dye solution on PCE of the fabricated devices were also studied. Different concentrations of the dye solution were prepared by serially diluting the dye in ethanol extract with ethanol, keeping the total volume as 12 mL (Table 4).

**Table 4:** Photovoltaic parameters of DSSCs with photoanodes sensitized by different concentrations of the dye under illumination intensity of 100 mWcm^–2^ with AM 1.5 filter.

Dye solution		J_SC_ (mAcm^–2^)	V_OC_ (V)	FF	η (%)
Volume of dye in ethanol extract (mL)	Volume of added ethanol (mL)				
12	–	0.72	0.58	0.64	0.27
9	3	0.81	0.60	0.64	0.31
6	6	0.82	0.62	0.66	0.34
3	9	0.66	0.61	0.62	0.25

**Figure 10:**
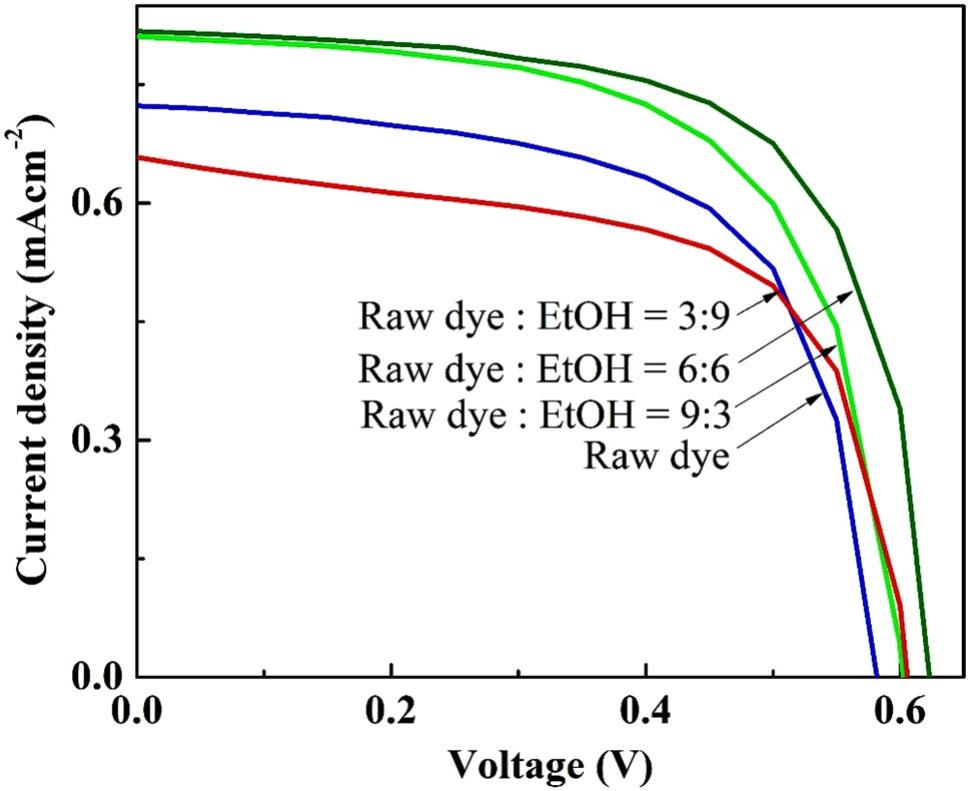
Photovoltaic performances of DSSCs with photoanodes sensitized by different concentrations of the dye under illumination intensity of 100 mWcm^–2^ with AM 1.5 filter.

The optical study of the prepared dye solutions confirmed that light absorption increases with increase in the dye concentration (Fig. S6). However, the PV performance of the corresponding devices was only slightly improved with the change in dye concentration (Fig. 10). When the natural dye extracted in ethanol was diluted to half of its initial concentration, a slight improvement in the PCE of the corresponding device was observed.

To investigate the influence of pH on the optical properties of the extracted natural dye, different volumes of 0.1 M HCl were separately added to the optimized dye solution (dye: ethanol = 6:6) and the resultant dye solutions were analyzed by UV–Visible spectroscopy. Subsequently, the TiO_2_ films were dipped in the said dye solutions at varied pH for 12 h, the corresponding DSSCs were fabricated, and their PV performances were evaluated.

**Figure 11:**
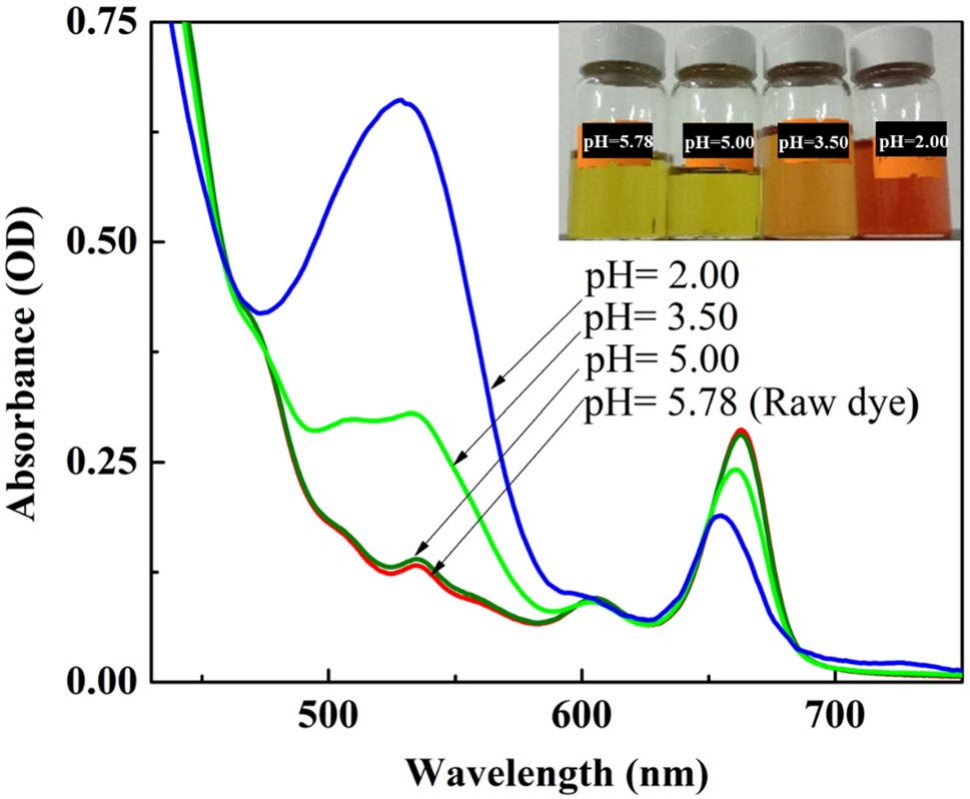
UV–Visible spectra of the dye solutions at different pH.

Figure 11 shows the UV–Visible absorption spectra of the dye solutions at four different pHs. The absorption spectrum of a dye reflects its optical transition probability between the ground state, the excited state and the solar energy range absorbed by the dye. The pH of the optimized dye solution was found to be 5.78. When the pH of the said dye solution was decreased step by step, the intensity of the peak corresponding to chlorophyll a showed a downward trend whereas the intensity of the peak responsible for anthocyanin exhibited the opposite trend in the UV–Visible spectra. It has been reported that exposure of chlorophyll a molecules to weak acids, oxygen or light accelerates their oxidation potential and results in the formation of numerous degradation products^36^ which could be attributed to its decreased peak intensity at low pH (acidic condition). Moreover, the colour of anthocyanin is sensitive to pH^37^ due to transformation of its molecular structure at different pH. In the present study, the natural dye extracted from *Mussaenda erythrophylla* flower in ethanol showed an absorption peak near 530 nm, corresponding to anthocyanin, at pH 5.78 and it became more intense and broader when the pH of the said dye solution was decreased to 2.00. It has been reported in the literature that protonation of anthocyanin molecules leading to the formation of red coloured flavylium salt occurs under acidic condition (inserted image in Fig. 11)^8^. As the optical properties of the dye solution varied with pH, devices were fabricated by employing the said dye solution at four different pH and their PV performances were investigated.

**Figure 12:**
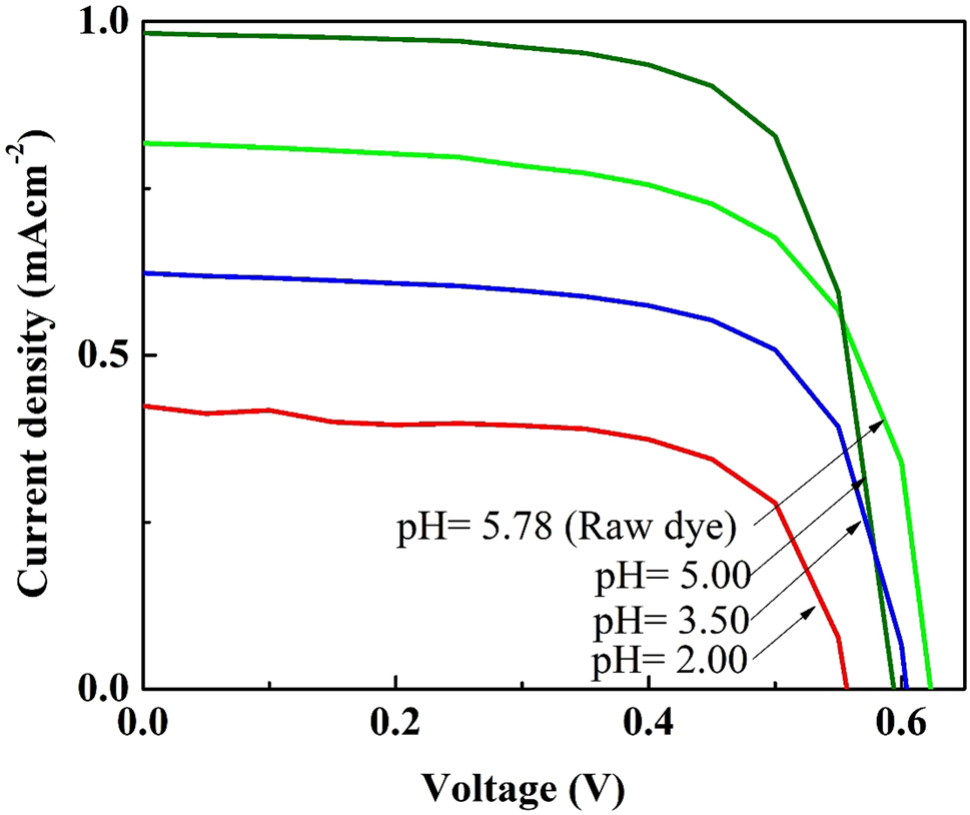
Photovoltaic performances of DSSCs with photoanodes sensitized by the natural dye solution at different pH under illumination intensity of 100 mWcm^–2^ with AM 1.5 filter.

As illustrated in Fig. 12 and Table 5, the optimum efficiency of 0.41% with the J_SC_ of 0.98 mAcm^–2^ and V_OC_ of 0.60 V was observed for the device fabricated with the natural dye extracted from *Mussaenda erythrophylla* flower in ethanol at pH 5.00. The investigation on the influence of pH on device performance revealed that when the pH was reduced from 5.78 to 5.00, the J_SC_ and PCE increased from 0.82 mAcm^–2^ and 0.34% to 0.98 mAcm^–2^ and 0.41% respectively. Hence, it is proposed that a small quantity of flavylium ions, the stable form of anthocyanin, are formed at pH 5.00, get firmly attached to the TiO_2_ and thereby enhance the PCE^3^. However, further decrease in pH reduces the device performance. It could be concluded that even though the natural dye extracted from the flowers of *Mussaenda erythrophylla* contains both chlorophyll a and anthocyanin, PCE of the device is largely influenced by the light absorption ability of chlorophyll a. Table 6 summarizes PV performances of DSSCs sensitized by natural dyes extracted from different plants.

**Table 5:** Photovoltaic parameters of DSSCs with photoanodes sensitized by the natural dye solution at different pH under illumination intensity of 100 mWcm^–2^ with AM 1.5 filter.

pH	J_SC_ (mAcm^–2^)	V_OC_ (V)	FF	η (%)
5.78	0.82	0.62	0.66	0.34
5.00	0.98	0.60	0.71	0.41
3.50	0.62	0.60	0.67	0.25
2.00	0.42	0.56	0.66	0.16

**Table 6:** A comparison of PV performances of DSSCs with natural dye sensitizers extracted from different plants.

Plant	J_SC_ (mAcm^–2^)	V_OC_ (V)	FF	η (%)	Ref
*Strobilanthes cusia* (leaves)	0.003	0.28	0.25	0.01	^38^
*Saraca asoca* (flowers)	0.29	0.51	0.65	0.09	^39^
*Indigofera tinctoria* (leaves)	0.37	0.48	0.63	0.11	^30^
*Lantana repens* (flowers)	0.45	0.69	0.34	0.12	^40^
Canna lily yellow (flowers)	0.43	0.56	0.40	0.12	^32^
Canna lily Red (flowers)	0.44	0.57	0.45	0.14	
*Codiaeum Variegatum* (leaves)	0.90	0.28	0.42	0.12	^41^
*Delonix Regia* (flowers)	1.18	0.32	0.40	0.17	
*Luffa cylindrica L* (flowers)	0.44	0.52	0.60	0.13	^42^
*Jabuticaba fruit* (fruits)	0.38	0.41	0.29	0.13	^43^
*Nymphaea pubescens Willd.* (flowers)	0.85	0.52	0.63	0.28	^44^
*Mussaenda erythrophylla* (flowers)	0.98	0.60	0.71	0.41	This work

The above comparison has revealed that the ethanol extract of *Mussaenda erythrophylla* flower is a promising candidate to serve as sensitizer in the DSSC application.

## Conclusion

A natural dye, extracted from the flowers of *Mussaenda erythrophylla* in ethanol and DI-water separately, was employed as a photosensitizer in DSSCs. The phytochemical analyses of the said dye confirmed the presence of many phytochemicals. The optical, structural and electrochemical characterization of the said dye and the dye coated TiO_2_ films revealed the presence of anthocyanin and chlorophyll a in the ethanol extract only. The DSSC fabricated with the optimized natural dye sensitizer in ethanol at pH 5.00 demonstrated the best PCE of 0.41% with 0.98 mAcm^–2^ of J_SC_.

### Supplementary Information

Supplementary Information.

## Data Availability

All data generated or analyzed during the present study are included in this article.
