# Supplementary material for: Natural sensitizer extracted from Mussaenda erythrophylla for dye-sensitized solar cell
Source: Sci Rep. 2023 Aug 24;13:13844. doi: 10.1038/s41598-023-40437-6 (PMC10449896; doi:10.1038/s41598-023-40437-6)
Supplement: Supplementary file 1 — Supplementary Information. [file 41598_2023_40437_MOESM1_ESM.docx]

**Natural sensitizer extracted from *Mussaenda erythrophylla* for dye-sensitized solar cell**

Tharmakularasa Rajaramanan^1,2,*^ , Fatemeh Heidari Gourji^1^, Yogenthiran Elilan^3^, Shivatharsiny Yohi ^3^, Meena Senthilnanthanan^3^, Punniamoorthy Ravirajan^2^ and Dhayalan Velauthapillai^1,*^

^1^Faculty of Engineering, Western Norway University of Applied Sciences, 5020 Bergen, Norway

^2^Clean Energy Research Laboratory (CERL), Department of Physics, University of Jaffna, Jaffna 40000, Sri Lanka

^3^Department of Chemistry, University of Jaffna, Jaffna 40000, Sri Lanka

Correspondence- [Dhayalan.Velauthapillai@hvl.no](mailto:Dhayalan.Velauthapillai@hvl.no); Tel.; +47-55-87711

[rramanan9@gmail.com](mailto:rramanan9@gmail.com) ; Tel.: +94775572984

**Supplementary Information**

Table S1: Phytochemical Tests

| Test | Procedure |
| --- | --- |
| Terpenoids | 5 mL of the extract was taken in a test tube and 2 mL of chloroform was added to it followed by the addition of 3 mL of conc. sulphuric acid. Formation of reddish-brown layer at the junction of two solutions confirms the presence of terpenoids. |
| Flavonoids | The extract was mixed with a few fragments of magnesium ribbon and conc. HCl was added drop wise. Appearance of pink scarlet color confirms the presence of flavonoids. |
| Glycosides | 5 mL of the extract was added to 2 mL of glacial acetic acid followed by the addition of 1 drop of ferric chloride solution and 1 mL of conc. sulphuric acid. Formation of a brown ring at the interface confirms the presence of glycosides. |
| Alkaloids (Hager’s test) | A few drops of 0.1% picric acid was added to 0.5 mL of the extract. Formation of yellow colour indicates the presence of alkaloids. |
| Phenols (Ferric chloride test) | A few drops of neutral ferric chloride (0.5%) solution was added to 0.5 mL of the extract. Formation of dark green colour indicates the presence of phenolic compounds. |
| Quinones | 1 mL of the extract was added to 1 mL of conc. H_2_SO_4_. Appearance of red colour indicates the presence of quinones. |
| Coumarins | 1 mL of the extract was added to 1 mL of 10% NaOH. Formation of yellow colour indicates the presence of coumarins. |

**
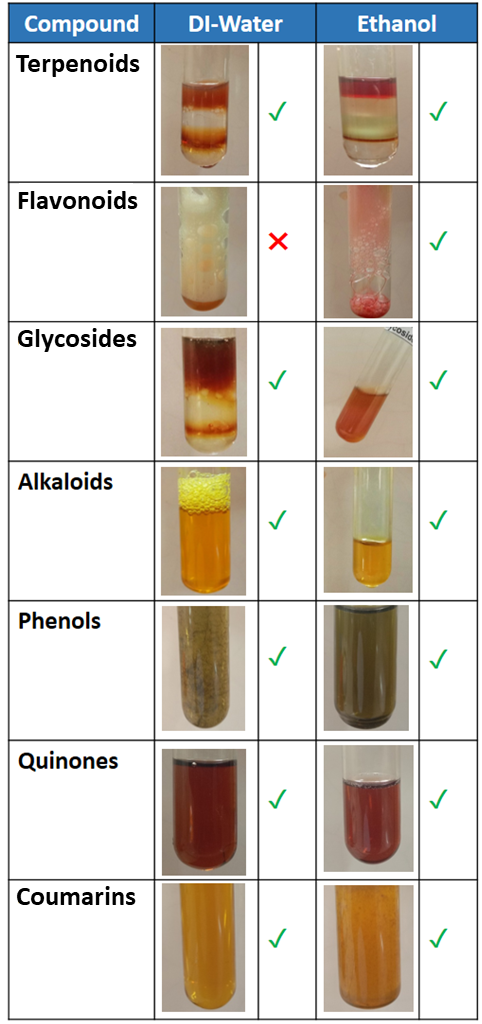
**

*Figure S1: Summary of the phytochemical analysis of the dye extracted in ethanol and DI-water*


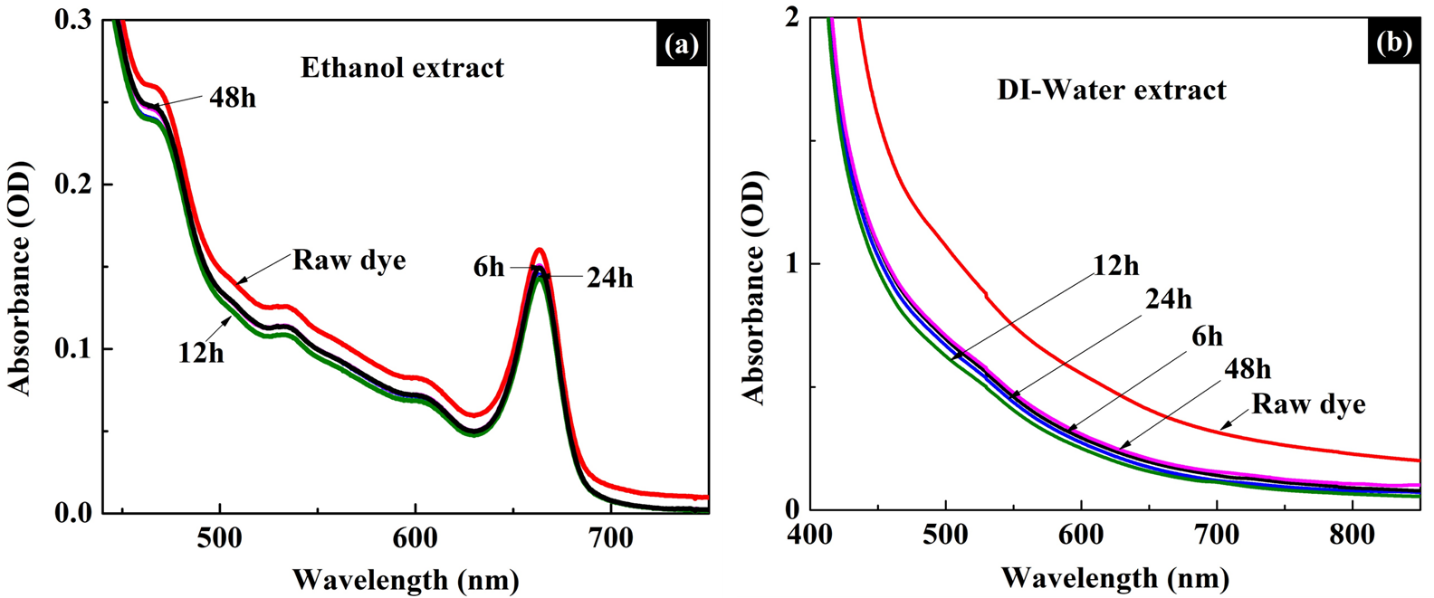


*Figure S2: Time-dependent UV-Visible spectra after dipping TiO_2_ film in (a) dye in ethanol extract (b) dye in DI-water extract*


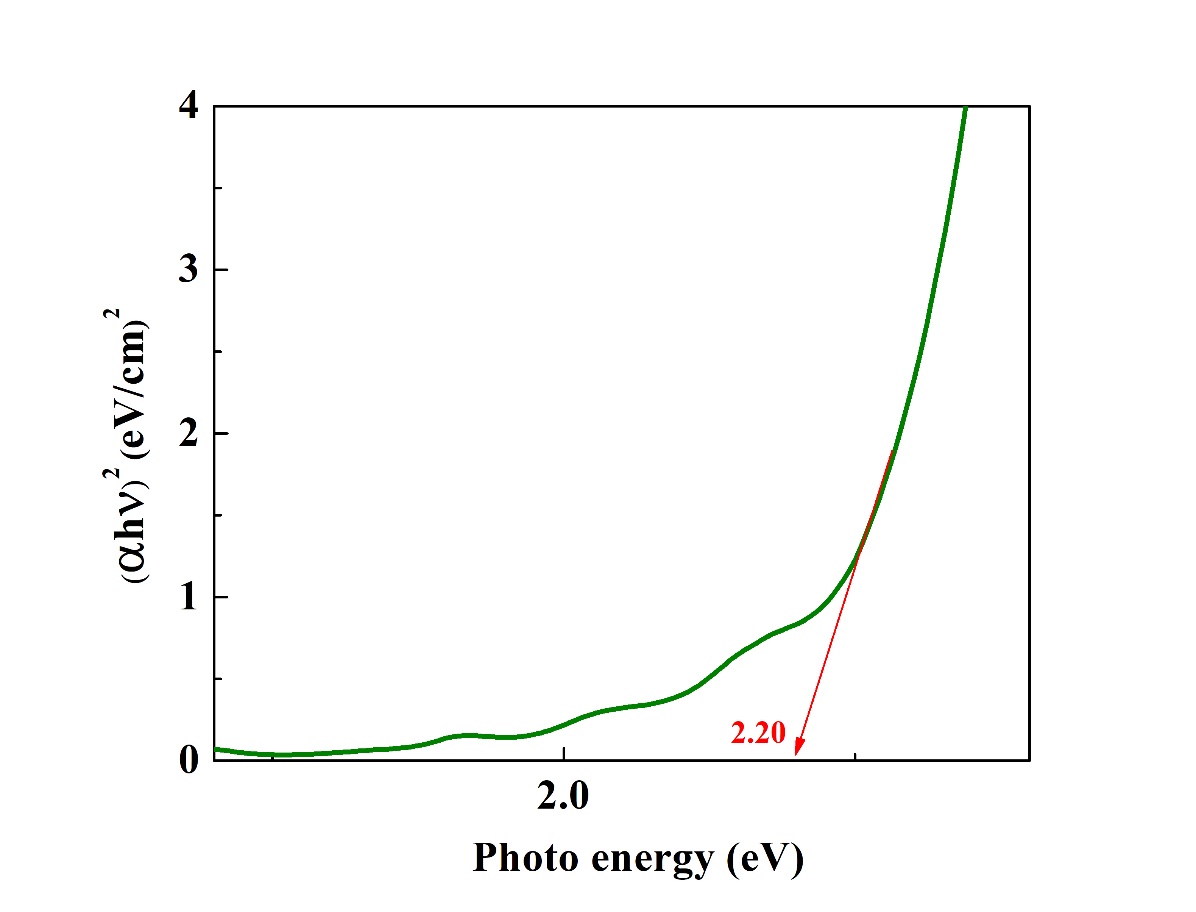


*Figure S3: Tauc’s plot of dye in ethanol extract*


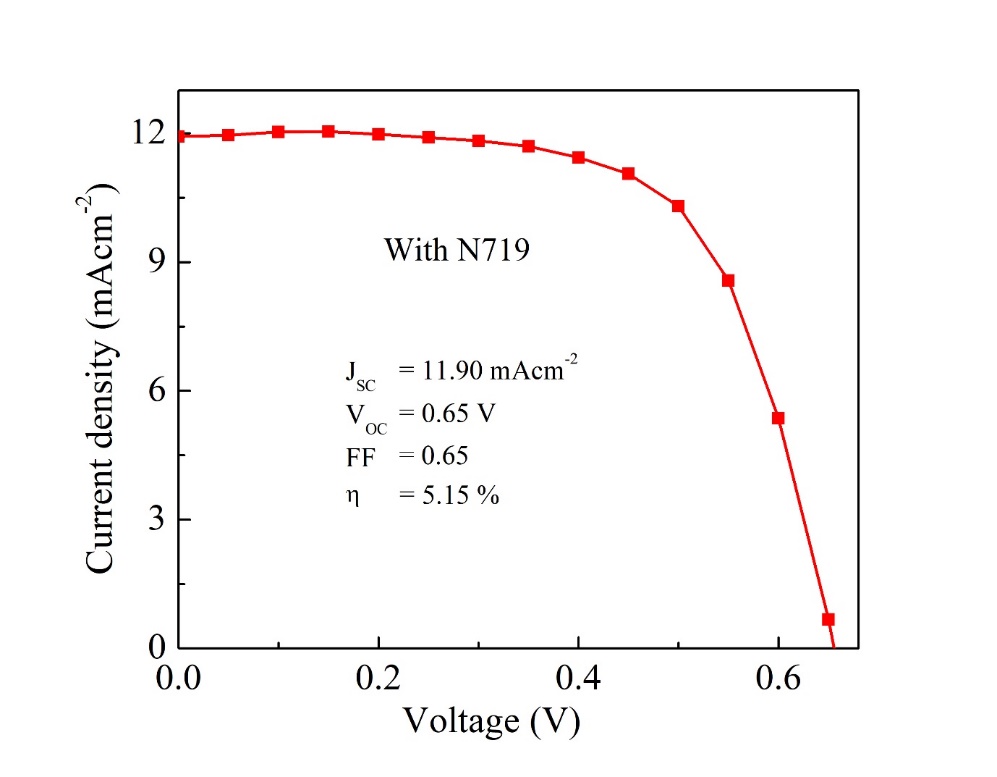


*Figure S4: Photovoltaic performance of DSSC with photoanode sensitized by N719 dye*


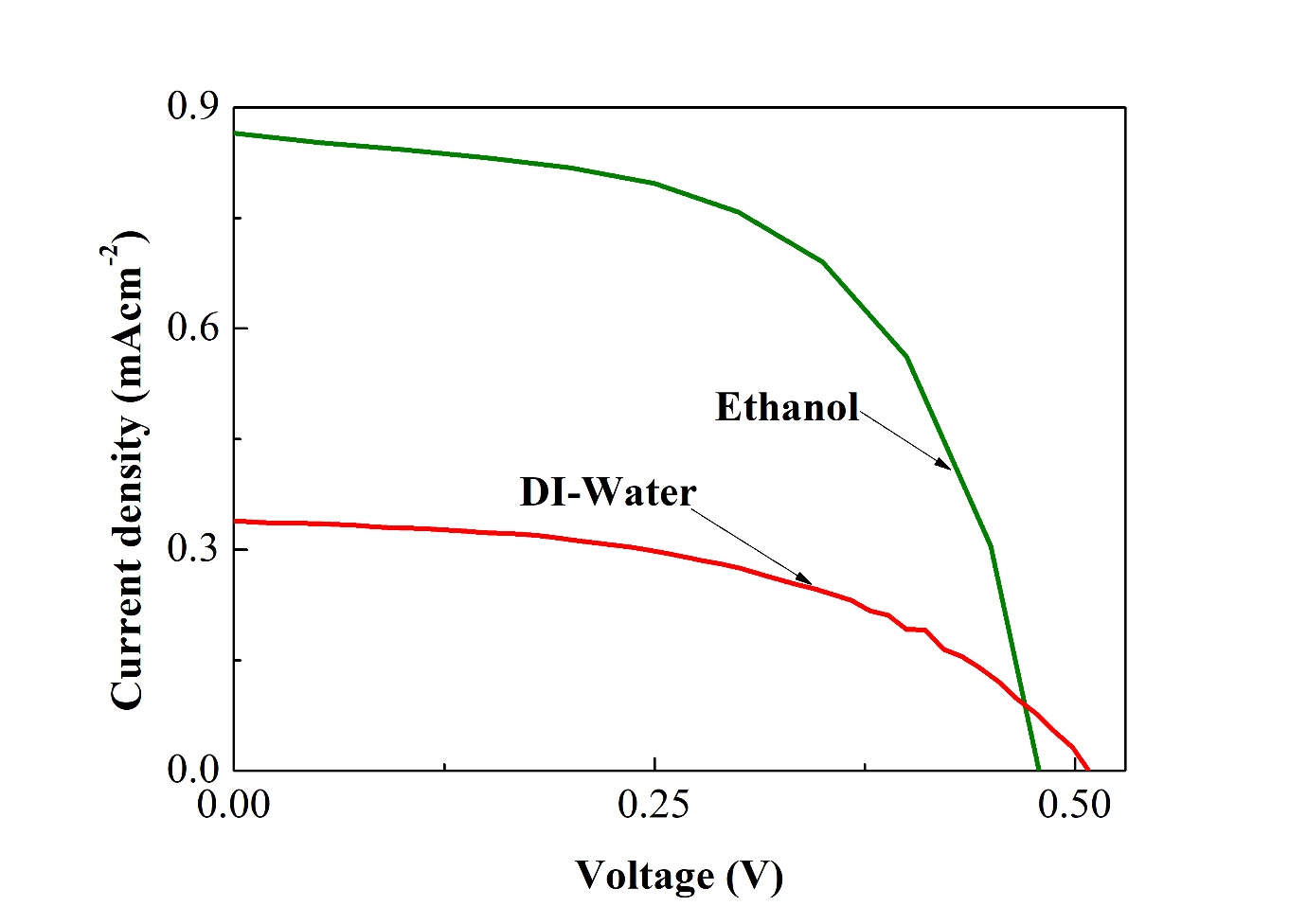


*Figure S5: Photovoltaic performances of DSSCs with photoanodes sensitized by dye in ethanol extract and dye in DI-water extract under illumination intensity of 100 mWcm^-2^ with AM 1.5 filter after 24 hours*

Table S2: Photovoltaic parameters of DSSCs with photoanodes sensitized by dye in ethanol extract and dye in DI-water extract under illumination intensity of 100 mWcm^-2^ with AM 1.5 filter after 24 hours

| **Solvent** | **J_SC_ (mAcm^-2^)** | **V_OC_ (V)** | **FF** | **η (%)** |
| --- | --- | --- | --- | --- |
| De-ionized (DI) water | 0.36 | 0.50 | 0.50 | 0.09 |
| Ethanol | 0.86 | 0.48 | 0.58 | 0.24 |


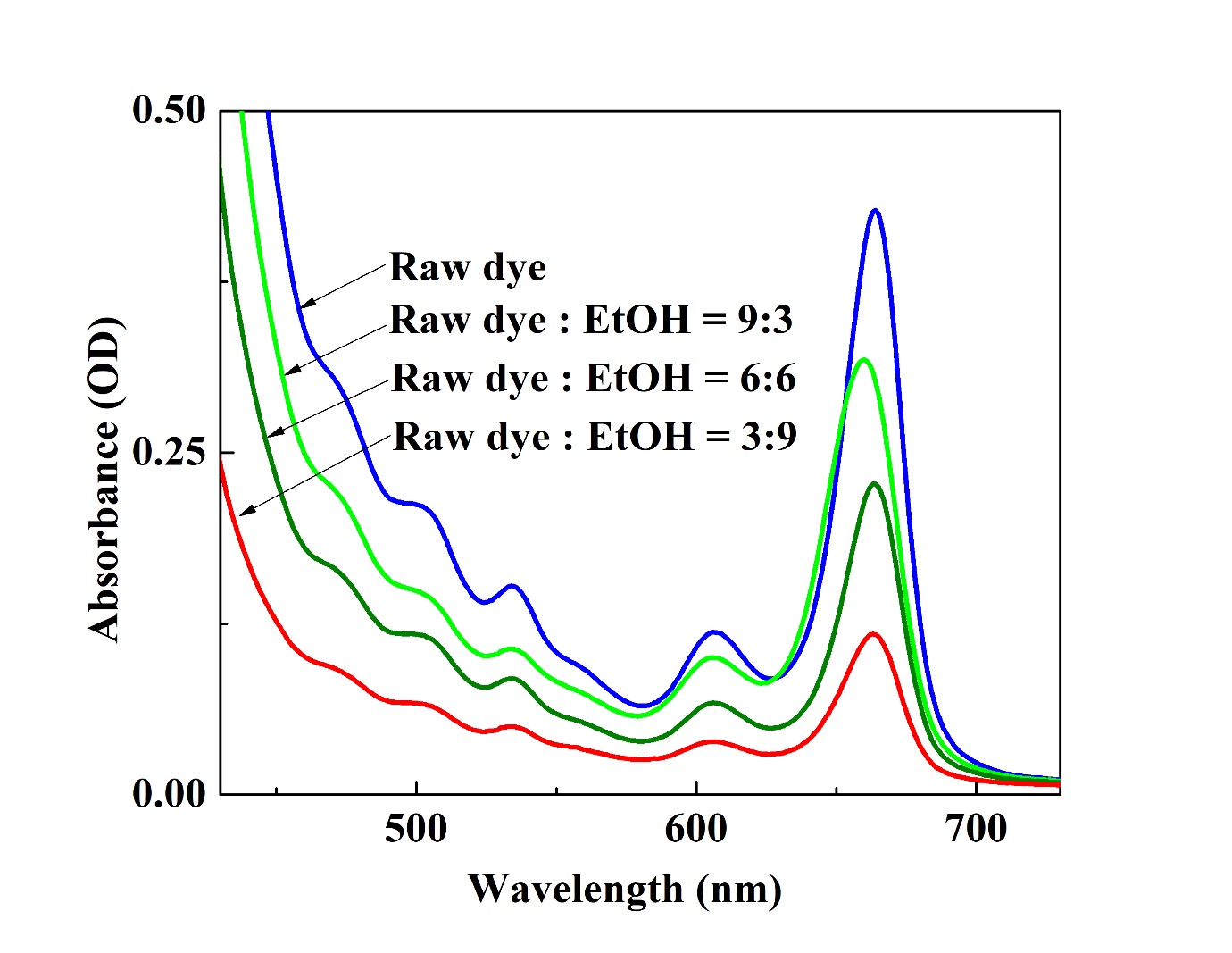


*Figure S6: UV-visible spectra of ethanol extract with different dye concentrations*
